# Glutamine alleviates radiation-induced intestinal injury in rats via the mTOR/Notch1 axis

**DOI:** 10.3389/fonc.2026.1735401

**Published:** 2026-02-04

**Authors:** Heng Lu, Xiangmin Ni, Xinyu Liang, Qian Bai, Haoyu Li, Mengran Shi, Yulong Jia, Jian Wang

**Affiliations:** 1 Department of Nutrition, The Second Affiliated Hospital, Army Military Medical University, Chongqing, China; 2 Xi’an Flying College of PLA Air Force, Xi’an, China; 3 Hematopoietic Acute Radiation Syndrome Medical and Pharmaceutical Basic Research Innovation Center, Ministry of Education of the People’s Republic of China, Chongqing, China

**Keywords:** glutamine, goblet cells, mTOR/Notch1 axis, MUC2, radiation-induced intestinal injury

## Abstract

**Introduction:**

Radiotherapy remains a principal modality for managing malignant pelvic tumors; nevertheless, it frequently induces radiation-associated intestinal injury (RIII), a debilitating complication that compromises intestinal barrier integrity. Glutamine (Gln) functions as an essential nutrient for maintaining and repairing the intestinal mucosa, yet its specific role in RIII and the mechanisms involved have not been clearly defined.

**Methods:**

In this study, we explored the protective potential and molecular mechanisms of Gln against RIII by employing both a localized abdominal irradiation rat model and irradiated HT-29 cell cultures.

**Results:**

Gln administration markedly mitigated intestinal shortening and mucosal injury, reduced the expression of pro-inflammatory cytokines, and restored the number of goblet cells (GCs). Furthermore, Gln treatment enhanced intestinal Lgr5 and Klf4 expression, suggesting protection of intestinal stem cells (ISCs) and facilitation of GC differentiation. Mechanistically, Gln activated the mTOR pathway and its downstream effectors S6K1 and 4E-BP1, while suppressing the radiation-induced overactivation of Notch1. Pharmacologic interventions using rapamycin and Jagged-1 further validated that Gln modulates the mTOR/Notch1 signaling cascade, leading to increased MUC2 expression and improved mucosal integrity.

**Discussion:**

Collectively, these results demonstrate that Gln confers robust protection against RIII by regulating the mTOR/Notch1 axis, alleviating Notch1 overactivation, and promoting GC differentiation. These findings provide valuable mechanistic insight and experimental support for Gln as a potential therapeutic agent to prevent or mitigate radiation-induced intestinal injury.

## 1 Introduction

Radiotherapy stands as a cornerstone in treating malignant pelvic tumors, significantly prolonging patient survival. However, its widespread application often results in severe complications due to radiation-induced damage to surrounding normal tissues, with radiation-induced intestinal injury (RIII) being the most prevalent, which manifests as urgency, frequent bowel movements, diarrhea, mucous stools, tenesmus, and anal pain (1). In severe cases, RIII can cause multi-organ failure or even death (2).

The pathogenesis of RIII involves complicated interaction of direct and indirect injury mechanisms, which are still not fully elucidated. Apoptosis is the major contributor to epithelial damage after irradiation. Radiation induces DNA damage in intestinal epithelial cells, triggering apoptosis and compromising the mucosal barrier. In addition, radiation also suppresses epithelial cell proliferation, which further impairs epithelial continuity and further aggravates disrupted intestinal mucosal barrier (3). This barrier is a complex defense network consisting of mucus, microbial, mechanical, and immune components. The mucus layer is primarily composed of the high-molecular-weight glycoprotein Mucin 2 (MUC2), which is the first line of protection against luminal irritants and pathogens. MUC2 is synthesized and secreted by goblet cells (GCs) (4, 5). These GCs derived from Lgr5^+^ intestinal stem cells (ISCs) in crypt, of which differentiation largely governed by Notch1 signaling (6, 7).

Previous study showed that exposure of radiation significantly reduced GC abundance, impaired intestinal functionality, simultaneously depleted ISCs, and weakened the regenerative potential of the mucosal barrier. Notably, promoting GC differentiation has been approved to restore epithelial integrity and attenuate mucosal damage (8). Therefore, the differentiation of ISC into GC is a promising treatment to counteract RIII.

Glutamine (Gln) is the most abundant free amino acid in the human body and plays a dominate role in maintaining intestinal mucosal metabolism and regeneration (9). After irradiation, both the physiological requirement of Gln is markedly elevated (10), suggesting that the essential contribution of Gln to protecting mucosal homeostasis. In addition, Gln is served as a major metabolite activating the mammalian target of rapamycin (mTOR). The mTOR is an important regulator involved gene transcription, translation, and protein synthesis, which is the fundament for cell growth, differentiation, and metabolic adaptation (11, 12).

Because RIII entails profound disturbances in cellular energy metabolism and biosynthetic activity—both tightly governed by mTOR—it becomes crucial to elucidate how Gln modulates mTOR signaling under such stress conditions. Previous *in vitro* evidence has shown that ISCs fail to develop characteristic bud-like structures in Gln-deficient environments, whereas supplementation restores their proliferative capacity (13). Complementary animal studies revealed that dietary Gln enhances intestinal MUC2 expression and increases GC abundance (14). Interestingly, Gln metabolism is also linked to Notch1 signaling, as inhibition of Notch1 via γ-secretase blockade increases cellular Gln consumption (15), though the underlying mechanisms remain incompletely defined.

Given that the relationship between Gln and radiation, the present study established localized abdominal irradiation rat model and irradiated HT-29 cells to investigate the protective role of Gln in RIII. The results demonstrated that Gln administration mitigated radiation induced intestinal damage, preserved epithelial integrity, and promoted ISC differentiation into GCs via mTOR/Notch1 signaling pathway. These fundings provide experimental evidence that Gln supplement is a potential therapy for RIII, which is safer and more effective application of radiotherapy in clinical oncology.

## 2 Materials and methods

### 2.1 Development and drug administration in a rat model for RIII

Fifteen male Sprague–Dawley rats (4–6 weeks old) were obtained from the Army Military Medical University (SCXK [YU] 2022-0011). Five rats were separated into a cage randomly and further received different treatments. There were three groups in this study, including control, IR, and Gln+IR groups. Rats in the Gln+IR group received daily intraperitoneal injections of Gln (Pricella, China) at a dose of 0.3 g/kg body weight for three consecutive days, while rats in the IR group received equivalent volumes of saline. On the third day, after the final injection, all rats were anesthetized by intraperitoneal injection with nembutal (40 mg/kg bodyweight), and positioned to expose the lower abdomen. The rest of the body was shielded with lead blocks. Rats in the IR and Gln+IR groups were exposed to a single localized abdominal irradiation of 15 Gy using a Co60 source (16). No treatment was performed in the control group. All rats were fed under standard conditions with ambient temperature at 22 ± 2°C, relative air humidity 40–70%, a 12/12 h light-dark cycle and free access to water and diet.

Relevant studies indicate that the peak of acute radiation-induced intestinal injury in rats occurs in 3–7 days (16, 17). In the current study,7 days after irradiation, rats were sacrificed by cervical dislocation after anesthesia with nembutal (40 mg/kg bodyweight), and colon samples were collected. The total length of colon was measured, and samples were immediately snap-frozen in liquid nitrogen for further analyses. During the post-irradiation period, fecal output, body weight, and survival were recorded. The disease activity index (DAI) was calculated according to the scoring criteria showed in **Table 1**.

**Table 1:** Disease activity index score.

Score	Loss of weight (%)	Fecal character	Occult blood/bloody stool
0	Normal	Normal	Negative
1	1-5	—	—
2	6-10	Diarrhea, blood sticking around the anus	Positive
3	11-15	—	—
4	>15	Diarrhea, blood sticking around the anus	Naked bloody stool

### 2.2 Hematoxylin and eosin and alcian blue-periodic acid Schiff staining of colon tissue

Colon specimens from each group were fixed in 4% paraformaldehyde (Servicebio, China), embedded in paraffin, and sectioned transversely. Deparaffinization was performed with xylene, followed by rehydration in graded alcohols. Staining was conducted using an HE Staining Kit (Solarbio, China) and an AB-PAS Staining Kit (Solarbio, China). Microscopic observations were made using an Olympus BX63 microscope (Japan). The histopathological scoring of the colon tissues was performed using the method described by Yoshio Araki et al. (18) (**Table 2**). GCs were counted in AB-PAS staining sections, and the number of GCs per crypt was statistically analyzed.

**Table 2:** Scoring criteria for colon histological lesions.

Score	(1) Surface epithelial loss (2) crypt destruction (3) inflammatory cell infiltration into the mucosa
0	no change
1	Localized and mild
2	localized and moderate
3	extensive and moderate
4	extensive and severe

### 2.3 Immunohistochemical staining

Paraffin sections were cut transversely, deparaffinized with xylene, washed with PBS, and underwent antigen retrieval using citrate buffer. The sections were then incubated with 3% hydrogen peroxide and blocked with goat serum. Sections were incubated overnight at 4°C with Lgr5 antibody and Klf4. Goat anti-rabbit secondary antibody was then applied, followed by incubation with diaminobenzidine (DAB) staining kit (ZSGB-BIO, China). Observations were made under an Olympus BX63 microscope (Japan), and positive staining was analyzed using Image J software. The antibodies used are listed in **Supplementary Table S1**.

### 2.4 Quantitative PCR

The total RNA of colon was extracted by TRIzol (TaKaRa, Japan). Subsequently, the cDNA was synthesized by reverse transcription kit (TaKaRa, Japan) according to the manufacturer’s instruction. To assess the mRNA expression levels of inflammation markers, qPCR was performed by Bio-Rad CFX Maestro system. GAPDH was served as a reference gene. Relative mRNA expression was calculated according to the 2^-ΔΔCt^ method. Primer sequences are showed in **Table 3**.

**Table 3:** Primer sequences.

Gene	Forward (5’-3’)	Reverse (5’-3’)
*IL-1β*	GACTTCACCATGGAACCCGT	CAGGGAGGGAAACACACGTT
*IL-6*	GACTTCACCATGGAACCCGT	CAGGGAGGGAAACACACGTT
*GAPDH*	TGTGAACGGATTTGGCCGTA	GATGGTGATGGGTTTCCCGT

### 2.5 Western blotting analysis

Colon tissues were homogenized, and the total protein was extracted using RIPA lysis buffer. Proteins were denatured with loading buffer at 95°C for 5 min. Then, the protein samples were separated via SDS-PAGE, and transferred onto PVDF membranes (Millipore, USA). The membranes were blocked with 5% skimmed milk at room temperature for 2 hours and incubated with primary antibodies overnight at 4°C. Subsequently, membranes were incubated with secondary antibodies at room temperature for 2 hours. The immunoblotting bands were measured through chemiluminescence detection system, and related expressions of proteins were quantified by ImageJ software. Antibodies used in this study are provided in **Supplementary Table S1**.

### 2.6 Cell modeling and treatment

To further investigate the mechanism by which Gln regulates GCs differentiation in RIII, an *in vitro* ISC radiation model was established using HT-29 cells. The HT-29 cell line, purchased from Wuhan Procell Life Science & Technology Co., Ltd., was cultured in McCoy’s 5A (Pricella, China) medium supplemented with 10% fetal bovine serum (Pricella, China) and 100 kU/L penicillin-streptomycin (Pricella, China), and incubated in a 5% CO2, 37°C incubator. HT-29 cells are capable of expressing various intestinal lineage-specific markers and differentiating along multiple lineages, thus are considered pluripotent, similar to ISC (19). Previous research indicates that, compared to the basal concentration of Gln at 2 mM (the standard concentration in the culture medium), a concentration of 4 mM Gln significantly demonstrates protective effects on HT-29 cells. Therefore, we selected 2 mM Gln as the baseline concentration group and 4 mM Gln as the experimental group concentration to study the effects of Gln on mTOR and Notch1 under radiation conditions.

### 2.7 Rapamycin intervention experiment

In this study, to specifically inhibit mTOR signaling, the HT-29 cells were treated with 20 ng/mL RAPA (Solarbio, China). Cells were divided into four groups: 2 mM Gln, 2 mM Gln + RAPA, 4 mM Gln, and 4 mM Gln + RAPA. 4 hours after treatments, all cells were exposed to radiation at dose of 8 Gy using a Co (60) source (20). After an additional 24 hours of culture, cells were collected for further analysis.

### 2.8 Jagged-1 intervention experiment

To investigate the role of mTOR/notch1 pathway on Gln radio-resistance effects in colon, HT-29 cells were treated with the 10 μmol/L Jagged-1 (MCE, USA) (21), which activates the Notch1 receptor. Four groups were set up: 2 mM Gln, 2 mM Gln + Jagged-1, 4 mM Gln, and 4 mM Gln + Jagged-1. 4 hours after treatments, all cells were exposed to radiation at dose of 8 Gy using a Co (60) source (20). After an additional 24 hours of culture, cells were collected for further analysis.

### 2.9 Immunofluorescence

HT-29 cells were plated in confocal culture dishes and grown to approximately 60% confluency. After 4 hours treatment, cells were fixed with 4% paraformaldehyde, permeabilized using 0.5% Triton X-100 (Beyotime, China). Then, cells were blocked with 10% goat serum (Beyotime, China) and incubated with anti-MUC2 antibody at 4°Covernight. Subsequently, cells were treated with a fluorescent secondary antibody at room temperature for 1 hour. After washing, cells were stained with anti-fade DAPI medium (Beyotime, China) and incubated in dark for 15 minutes. MUC2 expression was observed by confocal microscope.

### 2.10 Statistical analysis

Statistical analyses were performed using SPSS version 20, and graphs were generated with GraphPad Prism 8. Data are presented as mean ± SD. Comparisons between two groups were carried out by t-test, while one-way ANOVA was applied in multiple groups- comparison. When these assumptions were not met, non-parametric tests were applied. *P* < 0.05 was considered statistically significant.

## 3 Results

### 3.1 Preventive effects of Gln on RIII in rats

**Figure 1:**
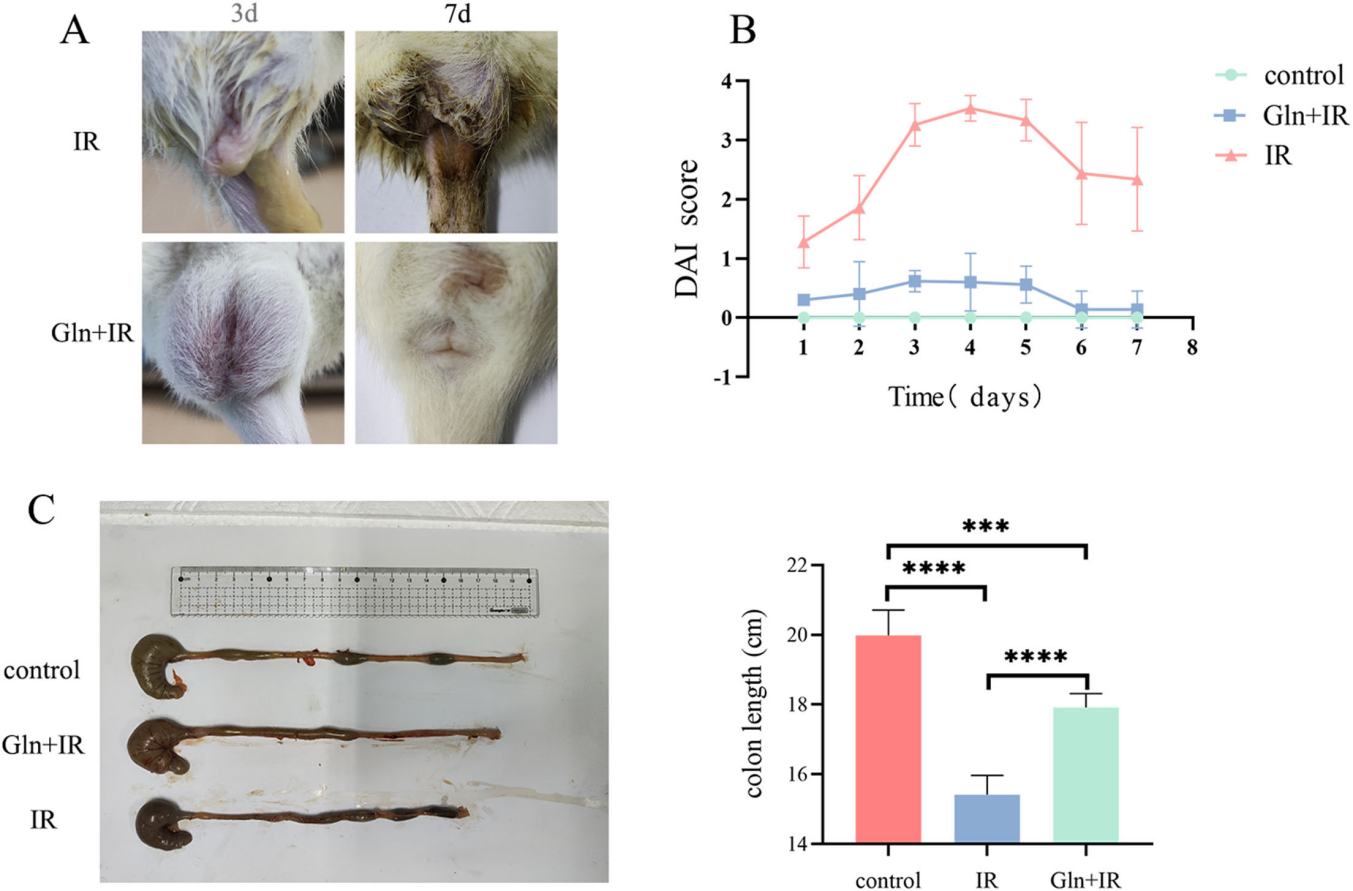
Effects of Gln on radiation-induced intestinal injury (RIII). **(A)** Representative images exhibiting diarrhea in IR and Gln+IR groups on day 3 and day7. **(B)** DAI in the experimental period. **(C)** Colon lengths at the end of experiment. Data are presented as mean ± SD (n = 5). One-way ANOVA was carried out followed by Tukey’s test. *P < 0.05, **P < 0.01, ***P < 0.001, ****P < 0.0001.

To explore the effects of Gln on RIII, classic intestinal injury markers were assessed after irradiation. Rats in the IR group exhibit loose stools and positive fecal occult blood, which were further progressed to diarrhea and rectal bleeding. However, no evident diarrhea or bleeding was found in rats in Gln+IR group, in which only loose stools and positive fecal occult blood were observed (**Figure 1A**). Additionally, the disease activity index (DAI) was used to evaluate the severity of the disease based on clinical symptoms such as weight loss, stool consistency, and bleeding. Compared with IR group, the DAI was significantly lower in Gln+IR group (**Figure 1B**). Importantly, colon length was significantly reduced in the IR group, whereas Gln significantly mitigated radiation-induced colon shortening (**Figure 1C**).

### 3.2 Gln mitigates intestinal inflammatory response

**Figure 2:**
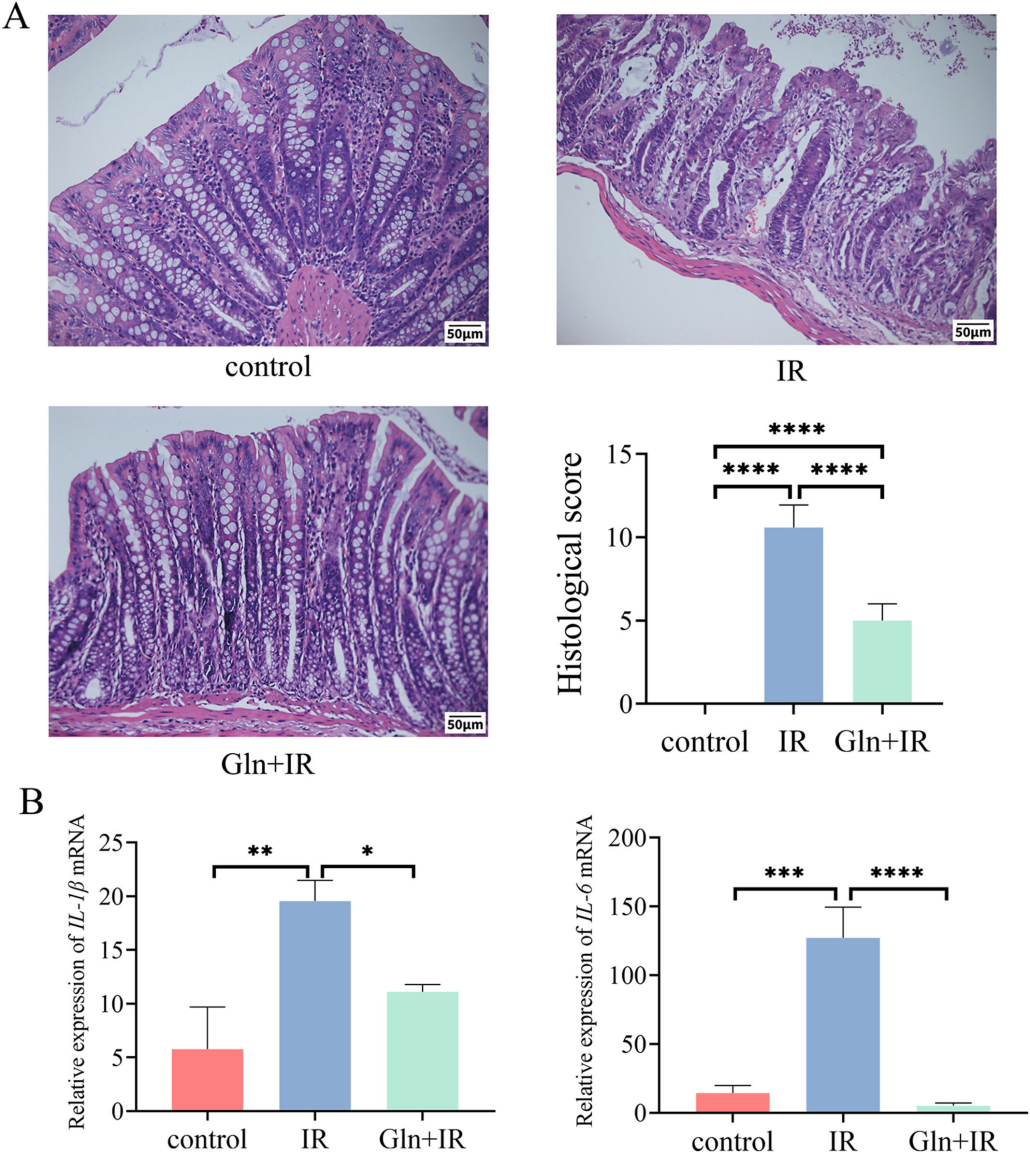
Effects of Gln on colonic histopathology and inflammation in irradiated rats. **(A)** HE staining (100×) and histopathological scores of rat colon. **(B)** Relative mRNA levels of IL-1β and IL-6. Data are presented as mean ± SD (n = 5). One-way ANOVA was carried out followed by Tukey’s test. *P < 0.05, **P < 0.01, ***P < 0.001, ****P < 0.0001.

To observe the structural damage of the intestines in RIII rats, HE staining of colon tissues was performed, which revealed that rats in the IR group exhibited extensive erosion, ulceration, inflammatory cell infiltration, and loss of normal crypt architecture. Pre-treatment with Gln significantly mitigated the radiation-induced damage in rat colons, as evidenced by the significantly lower histological scores in the Gln+IR group compared to the IR group (**Figure 2A**). Inflammatory response is one of the key features of colonic injury. The results showed that transcription levels of inflammatory cytokines *IL-1β* and *IL-6* were significantly higher in the IR group compared to the control group, whereas these levels were significantly reduced in the Gln+IR group compared to the IR group (**Figure 2B**).

### 3.3 Effects of Gln on rat colon GCs

**Figure 3:**
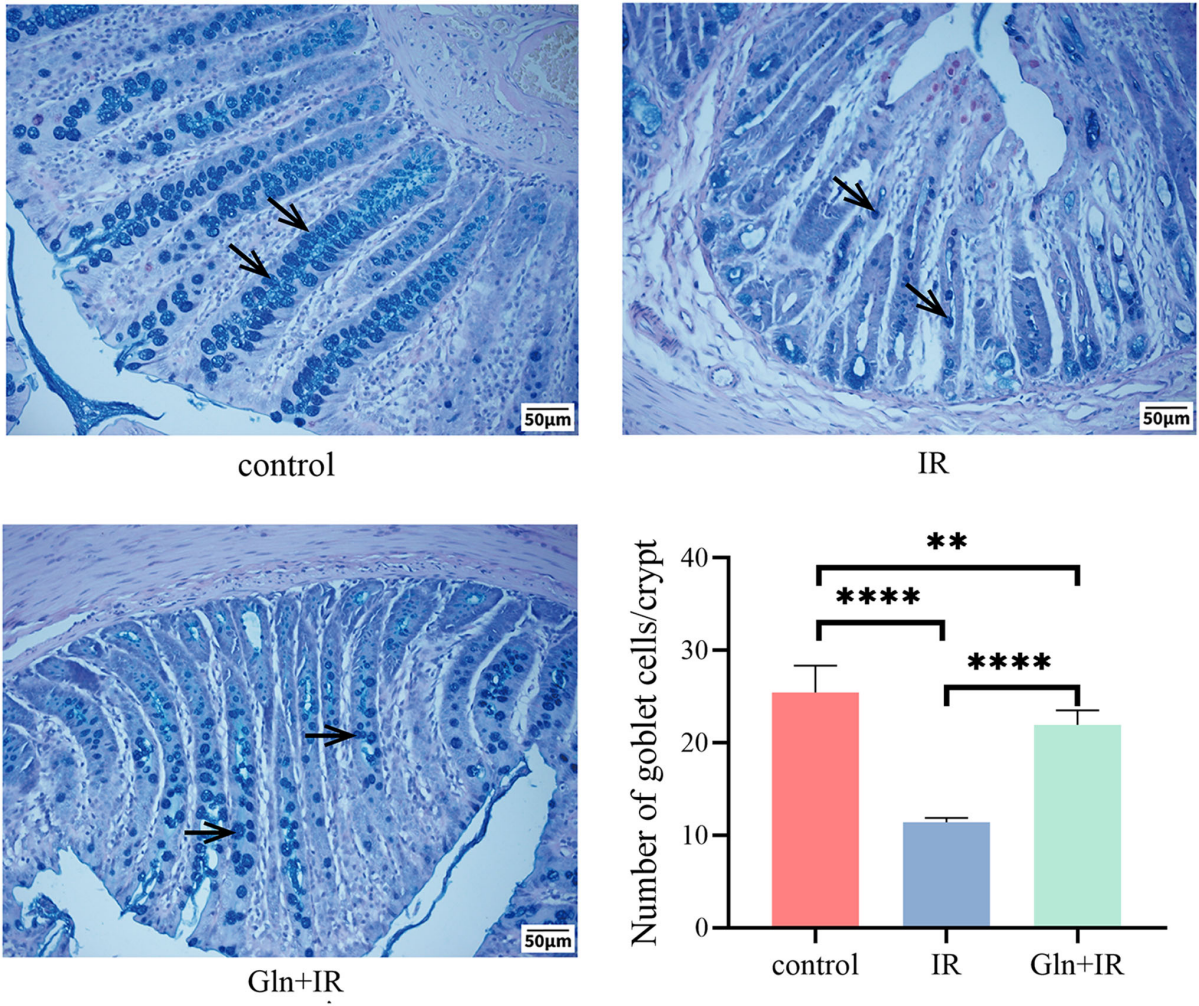
Effects of Gln on goblet cell numbers in rat colon tissue. AB-PAS staining was performed to visualize GC in colon sections, arrows indicating GCs. The number of GCs in per crypt. Data are presented as mean ± SD (n = 3). One-way ANOVA was carried out followed by Tukey’s test. *P < 0.05, **P < 0.01, ***P < 0.001, ****P < 0.0001.

AB-PAS staining was used to assess mucus production and evaluate the integrity of the intestinal barrier, particularly by highlighting goblet cells (GCs) involved in mucus secretion. The results showed GCs were significantly decreased in the IR group compared to the control, which was approaching depletion. In contrast, the Gln significantly increased colonic GCs (**Figure 3**). These results indicated that radiation induced severe loss of intestinal GCs in rats, whereas intraperitoneal Gln significantly attenuates radiation-induced GC depletion.

### 3.4 Effects of Gln on Lgr5 and Klf4 expression in rat colon

**Figure 4:**
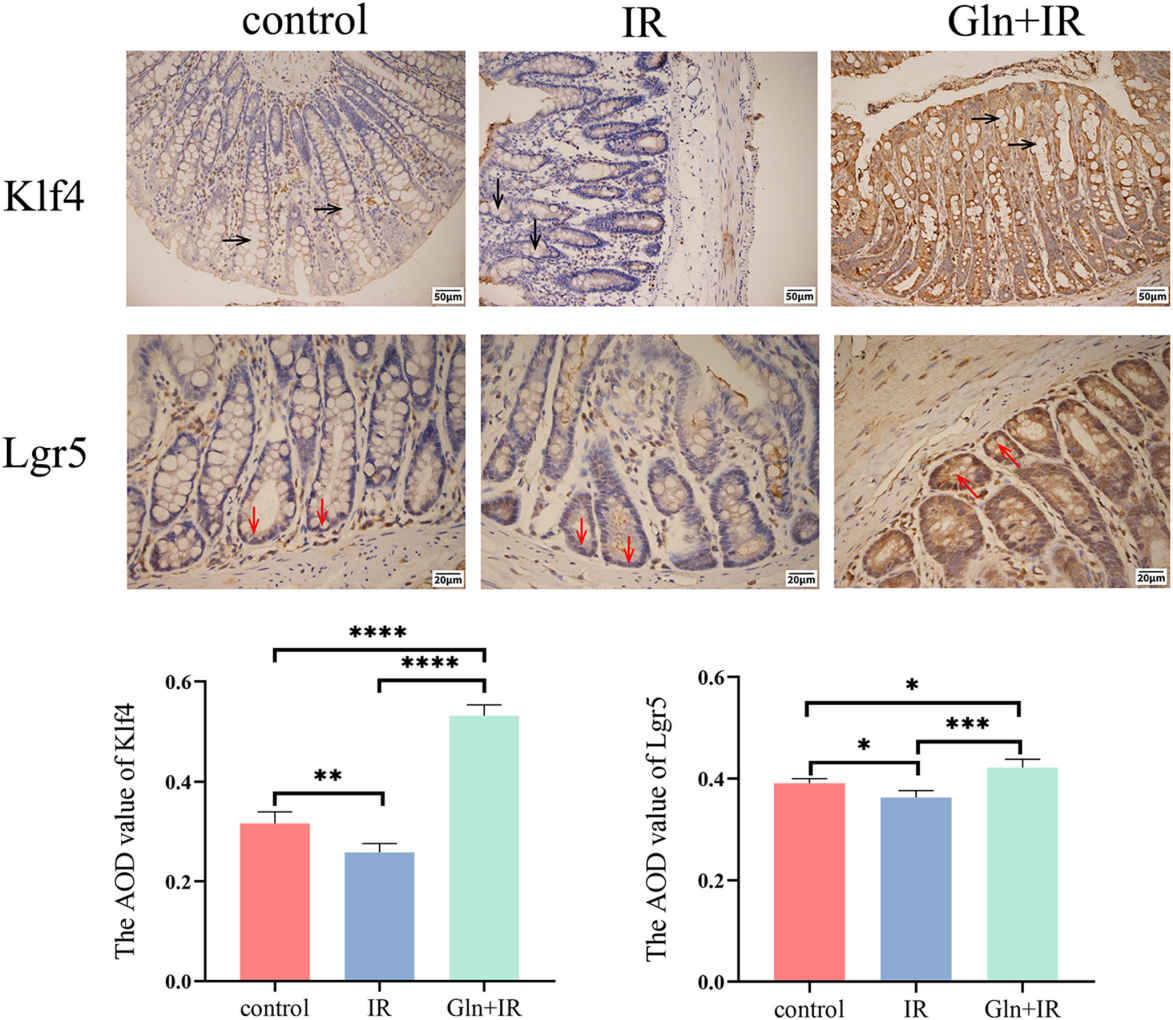
Effects of Gln on Klf4 and Lgr5 expression in rat colon tissue. Immunohistochemical staining of Klf4 and Lgr5 in colon sections. Black arrows indicate Klf4 (100×), and red arrow indicate Lgr5 (200×). The average optical density (AOD) of the stained sections was quantified using ImageJ software. Data are presented as mean ± SD (n = 3). One-way ANOVA was carried out followed by Tukey’s test. *P < 0.05, **P < 0.01, ***P < 0.001, ****P < 0.0001.

To investigate the effects of Gln on intestinal stem cells (ISCs) and goblet cell (GC) maturation, immunohistochemical analysis of colon tissues was performed. The results confirm that the expression of Lgr5, a marker of ISCs, is significantly higher in the Gln+IR group compared to the IR group, indicating that pre-administration of Gln protects ISCs. Additionally, the expression of Klf4, a marker of GC maturation, is significantly increased in the Gln+IR group, suggesting enhanced goblet cell differentiation and maturation (**Figure 4**).

### 3.5 Effects of Gln on mTOR signaling pathway and Notch1 in rat intestines

**Figure 5:**
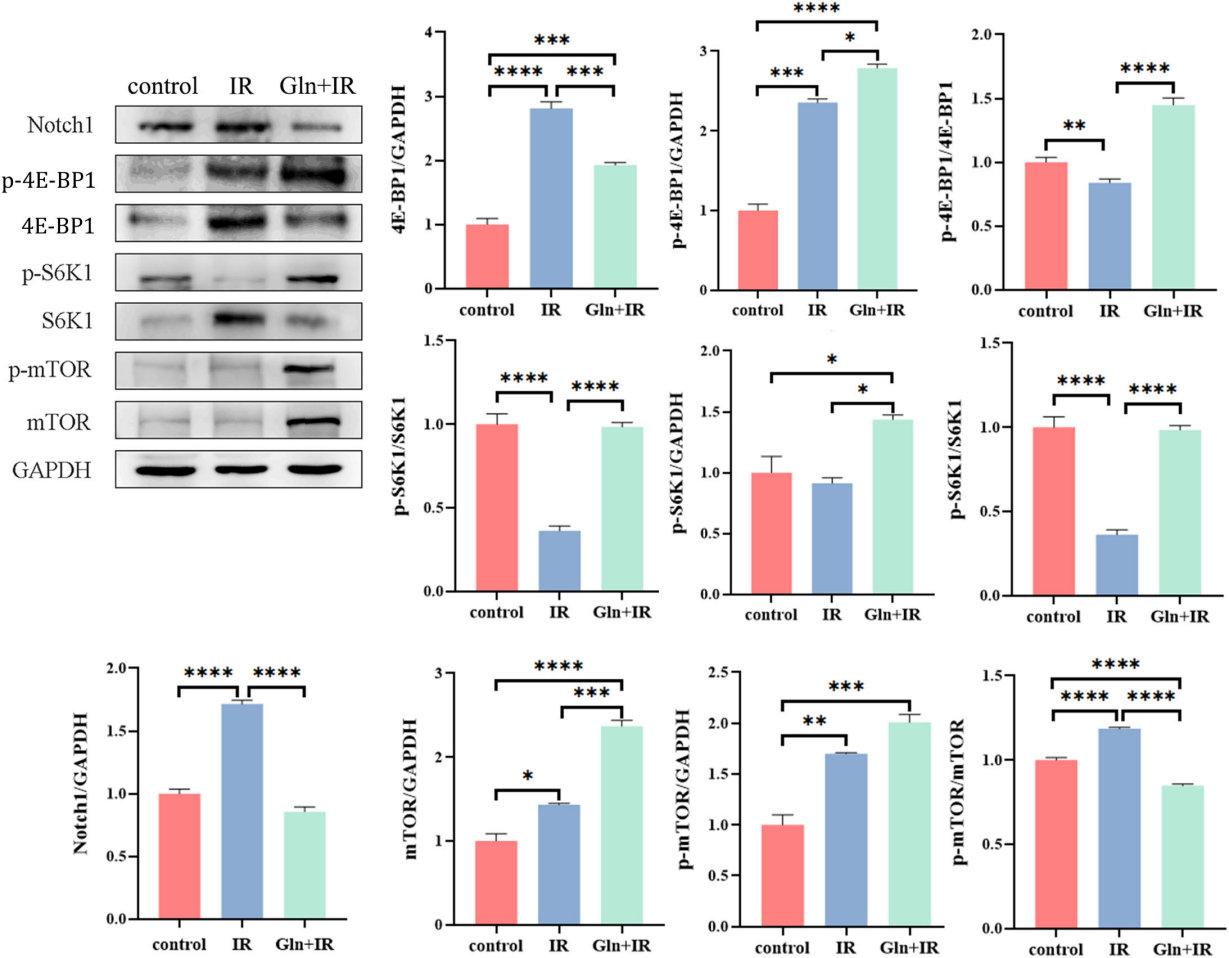
Effects of Gln on the mTOR signaling pathway and Notch1 in rat colon tissue. Western blotting was performed to assess the expression of mTOR, p-mTOR, S6K1, phosphorylated S6K1 (p-S6K1), 4E-BP1, phosphorylated 4E-BP1 (p-4E-BP1), Notch1, and GAPDH in colon tissues. The relative expressions of proteins were quantified by ImageJ software. Data are presented as mean ± SD (n = 3). One-way ANOVA was carried out followed by Tukey’s test. *P < 0.05, **P < 0.01, ***P < 0.001, ****P < 0.0001.

Notch1 and mTOR are key signaling pathways involved in cellular growth, differentiation, and response to stress, making them critical targets in radiation-induced injury and recovery. To investigate the effects of Gln on the mTOR signaling pathway and Notch1 expression in rat intestines, western blot analysis was performed. The results showed that Notch1 expression in the colon was significantly elevated in the IR group compared to the control, but it was significantly reduced in the Gln+IR group. These findings suggest that intraperitoneal Gln administration inhibits radiation-induced Notch1 overexpression in the rat colon. Additionally, Gln significantly increased the phosphorylation of mTOR and its downstream targets, S6K1 and 4E-BP1, compared with the IR group, indicating that Gln activates the mTOR signaling pathway. Phosphorylation of S6K1 and 4E-BP1 plays crucial roles in promoting protein synthesis, cell growth, and survival. Specifically, phosphorylated S6K1 enhances ribosomal biogenesis and translation initiation, while 4E-BP1 phosphorylation relieves its inhibition on eukaryotic translation initiation factor 4E (eIF4E), thereby further promoting protein synthesis. These findings suggest that Gln not only activates the mTOR pathway but also supports cellular processes essential for repair and regeneration following radiation-induced damage (**Figure 5**).

### 3.6 Effects of mTOR inhibition by RAPA on Notch1 and MUC2 expression

**Figure 6:**
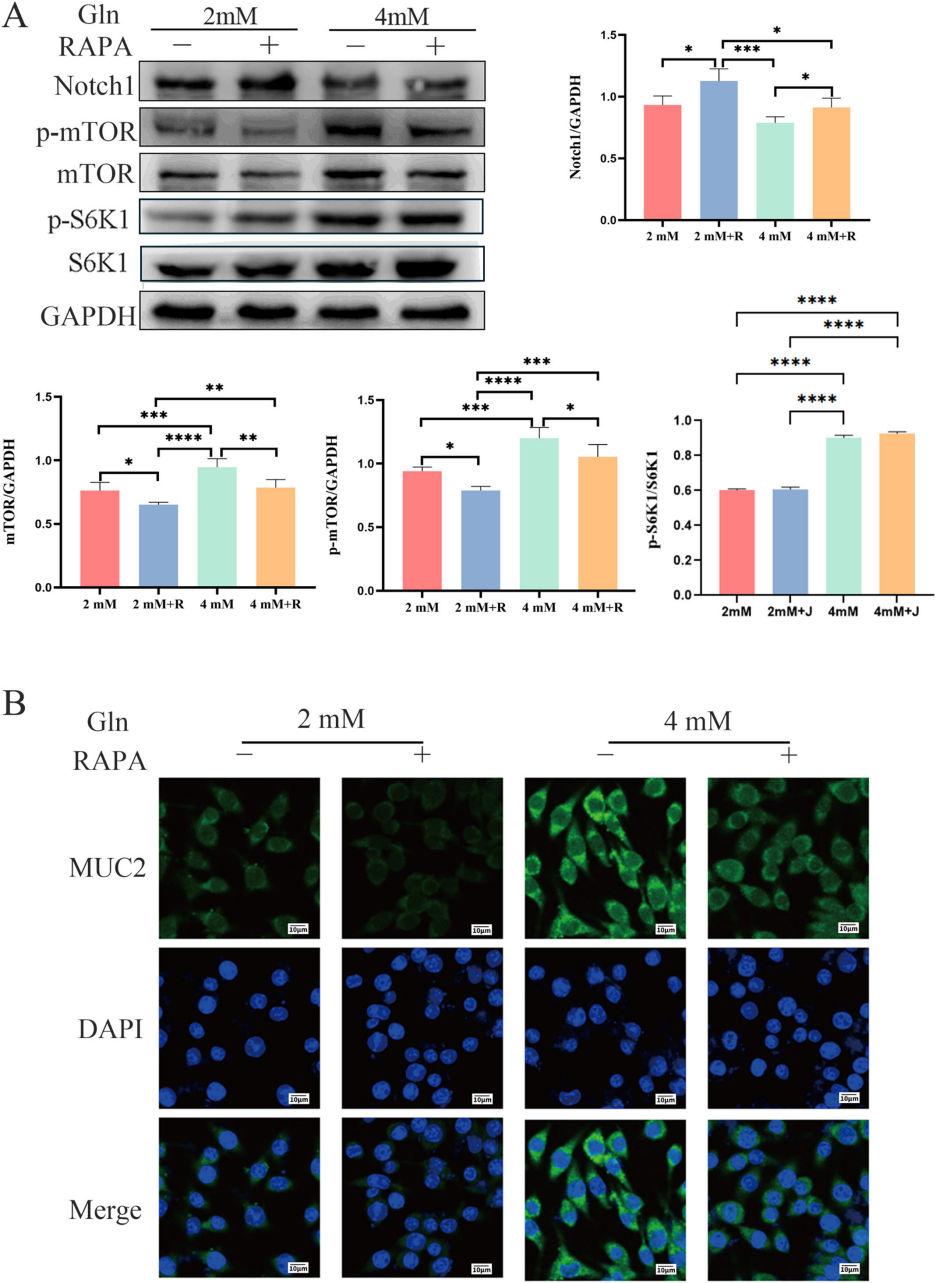
Effects of Gln and RAPA on the mTOR/Notch1 axis and MUC2 expression in HT-29 cells. **(A)** The expression of mTOR, p-mTOR, Notch1, and GAPDH. **(B)** Immunofluorescence analysis of MUC2 in HT-29 cells (800×). Data are presented as mean ± SD (n = 3). One-way ANOVA was carried out followed by Tukey’s test. *P < 0.05, **P < 0.01, ***P < 0.001, ****P < 0.0001.

To examine the effects of mTOR inhibition on Notch1 and MUC2 expression, mTOR was pharmacologically inhibited by RAPA in HT-29 cells. The results show that in the 4 mM group, the expression and phosphorylation levels of mTOR are significantly higher than in the 2 mM group, accompanied by a downward trend in Notch1 expression and upregulated MUC2 expression. After adding RAPA, both the 2 mM+R and 4 mM+R groups exhibit lower mTOR expression and phosphorylation levels compared to the 2 mM and 4 mM groups, respectively. Notch1 expression in the 2 mM+R and 4 mM+R groups is higher than in the 2 mM and 4 mM groups, respectively, while MUC2 expression is inhibited in HT-29 cells. These findings suggest that while Gln activates the mTOR signaling pathway and suppresses Notch1 expression, inhibiting mTOR with RAPA can restore Notch1 expression and inhibit MUC2 expression (**Figure 6**).

### 3.7 Effects of Notch1 activation by Jagged-1 on mTOR and MUC2 expression

**Figure 7:**
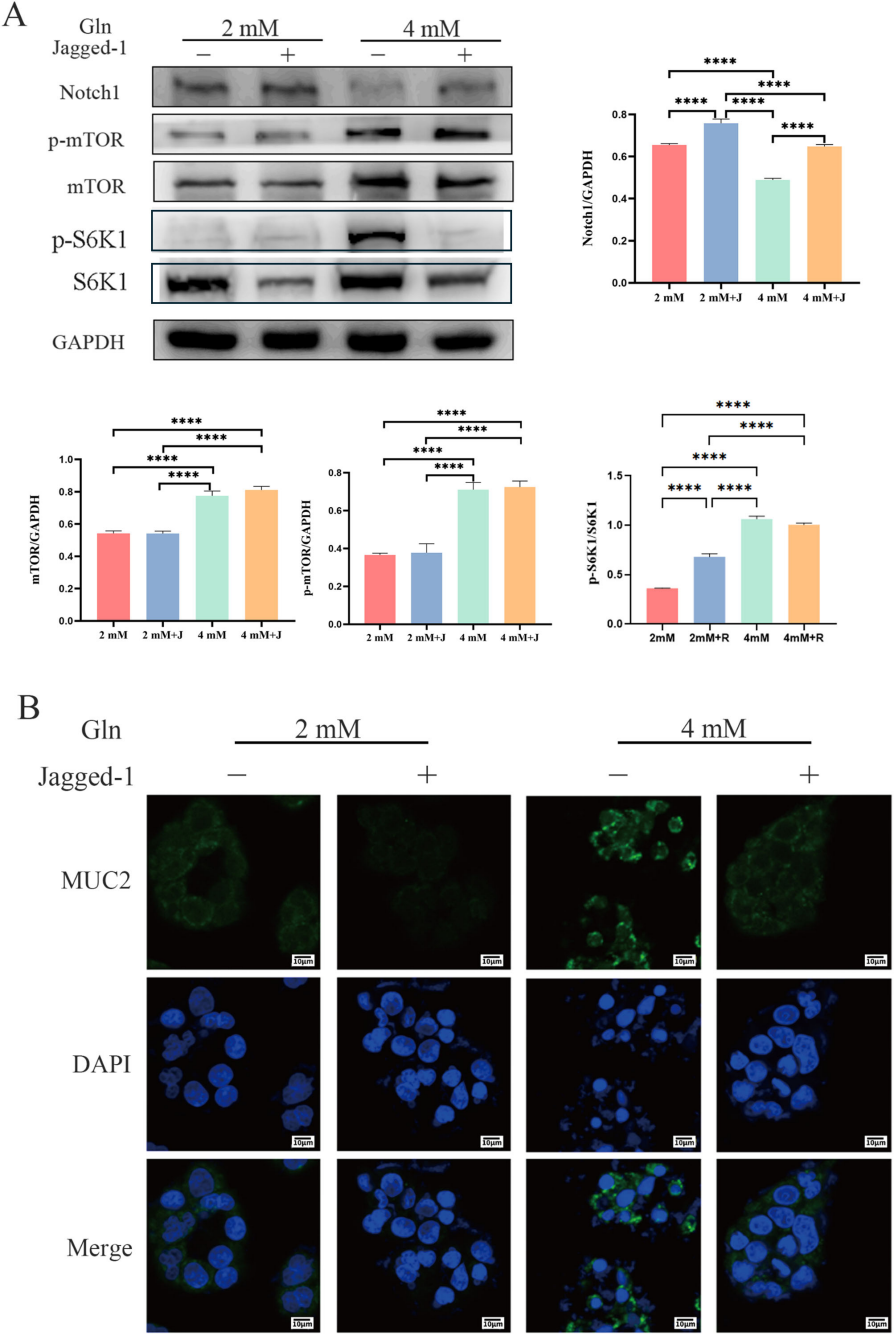
Effects of Gln and Jagged-1 on the mTOR/Notch1 axis and MUC2 expression in HT-29 cells. **(A)** The expression of mTOR, p-mTOR, Notch1, and GAPDH. **(B)** Immunofluorescence analysis of MUC2 in HT-29 cells (800×). Data are presented as mean ± SD (n = 3). One-way ANOVA was carried out followed by Tukey’s test. *P < 0.05, **P < 0.01, ***P < 0.001, ****P < 0.0001.

To explore the role of the Notch1/mTOR signaling pathway in regulating the intestinal barrier, HT-29 cells were treated with Jagged-1, a Notch1 agonist. The expression of Notch1 was significantly enhanced in both 2 mM+J and 4 mM+J groups compared with the 2 mM and 4 mM groups, respectively. In addition, MUC2 expressions in both 2 mM+J and 4 mM+J were significantly downregulated. However, no significant changes were observed in mTOR expression or phosphorylation levels. Additionally, Notch1 expression in 4 mM+J group was lower than that in 2 mM+J group. These results suggest that activation of Notch1 does not markedly affect mTOR expression or phosphorylation, while Gln can inhibit Jagged-1–induced Notch1 activation (**Figure 7**).

## 4 Discussion

With the growing incidence of pelvic malignancies and the broader clinical adoption of radiotherapy, RIII has emerged as a major complication limiting treatment efficacy. To date, there remains no standardized therapeutic protocol for RIII, and clinical management continues to depend largely on multidisciplinary care. Considering that the intestine is the primary organ responsible for nutrient absorption and transport, maintaining its structural and functional integrity is crucial for preventing or attenuating post-radiation enteritis. Existing nutritional support strategies for RIII are primarily reactive, focusing on replenishing energy and nutrients after intestinal damage has occurred. In contrast, proactive nutritional interventions—particularly those emphasizing precise amino acid supplementation to prevent or minimize radiation-induced injury—have received limited attention. Gln, as the principal energy substrate for intestinal epithelial cells, supports mucosal renewal and barrier integrity. Because radiation exposure rapidly depletes free Gln pools, early supplementation before irradiation may offer superior protective benefits, a concept supported by the findings of this study. Our data show that pre-administration of Gln in rats effectively mitigates radiation-induced intestinal pathology, reducing diarrhea, rectal bleeding, weight loss, colon shortening, inflammatory cytokine elevation, mucosal erosion, and GC depletion.

The doses of Gln used typically range from 0.25 to 1.0 g/kg per day (22–24). The clinical dosage of injectable Gln for adults is typically between 0.3 and 0.5 g/kg/day (25). Therefore, we selected the lowest dose of 0.3 g/kg/day as the preventive dose. The ability of intestinal epithelial cells to regenerate is essential for maintaining barrier integrity and nutrient absorption. ISCs are central to this regenerative process, possessing remarkable plasticity that enables both self-renewal and differentiation into multiple specialized cell types required for intestinal homeostasis (26). These processes are tightly regulated by several key signaling cascades, including the Notch pathway (27). Under physiological conditions, ISCs give rise to absorptive enterocytes, mucus-producing GCs, hormone-secreting enteroendocrine cells, and Paneth cells that release antimicrobial peptides (28).

Along the intestinal tract, GC density increases progressively from the duodenum toward the distal colon (29). During differentiation and maturation, GCs migrate upward along the crypt–villus axis before being exfoliated into the lumen (30, 31). This developmental trajectory is coordinated by multiple transcription factors that define GC identity and function (32, 33). Recent single-cell RNA sequencing analyses have identified a rare ISC subset—termed “revival stem cells”—that remains quiescent under normal conditions but becomes activated following tissue injury to regenerate all major intestinal epithelial lineages, including Lgr5^+^ cells (34). Our present data suggest that Gln administration preserves Lgr5^+^ ISCs, although whether this effect involves activation of revival stem cells warrants further investigation.

The intestinal mucus layer represents a vital defensive barrier that separates epithelial cells from luminal microorganisms and toxic contents (35). Under normal physiological conditions, this layer limits bacterial contact with the epithelium, while GCs actively participate in bacterial clearance, thereby preserving epithelial integrity. Loss or depletion of GCs disrupts this barrier, allowing microbial translocation and aggravating intestinal inflammation (36). In this study, we observed that Gln administration preserved GC abundance following radiation exposure. Immunohistochemical staining revealed elevated expression of Klf4—a key transcription factor driving GC differentiation—indicating that intraperitoneal Gln enhances GC maturation *in vivo*. Complementary *in vitro* data further demonstrated that Gln supplementation increases MUC2 expression in irradiated cells, reinforcing its role in maintaining mucosal defense.

Recent studies have shown that intestinal stem cell (ISC) behavior is shaped by both intrinsic metabolic circuits and extrinsic nutritional factors such as lipids, carbohydrates, and vitamins. Among these nutrients, Gln plays a pivotal role by activating the mammalian target of rapamycin (mTOR), a central nutrient-sensing kinase that integrates metabolic and growth signals to regulate cell proliferation, differentiation, and survival (37–40). Supplementation with Gln has been reported to selectively stimulate intestinal mTOR signaling, promote epithelial regeneration, and prevent crypt atrophy under nutrient-deficient conditions (12).

Intriguingly, mTOR signaling exhibits functional cross-talk with the Notch1 pathway. Mungamuri et al. (41) first demonstrated that mTOR activation is indispensable for Notch1-driven oncogenic signaling in breast cancer and T-cell acute lymphoblastic leukemia, and that mTOR inhibition can attenuate this effect. Other disease models have suggested the reverse interaction, where Notch1 downregulation dampens mTOR activity, implying a bidirectional regulatory loop (42, 43). Our findings provide *in vivo* confirmation of this relationship: Gln activates mTOR while suppressing radiation-induced Notch1 overexpression in the rat RIII model. Consistent with these results, *in vitro* experiments revealed that mTOR acts upstream of Notch1, modulating its expression and thereby influencing goblet cell differentiation and mucosal restoration.

*In vitro* experiments, even though Gln was proved to alleviated damage by radiation, the cellular model used in this work cannot fully represent the complex regenerative dynamics of ISCs under irradiation. Lineage-tracing or organoid-based systems is necessary to explore ISC fate in future study. Moreover, given that the intestinal epithelium comprises multiple cell lineages, GC differentiation in this study only clarified part of protective effects of Gln. Further research about influences of Gln on other epithelial subsets should be investigated to provide a comprehensive understanding of its role in mitigating RIII.

## 5 Conclusions

In summary, the *in vivo* and *in vitro* studies demonstrate that radiation induces Notch1 overactivation, which disrupts the establishment and renewal of the intestinal mucosal barrier. Gln mitigates this effect via the mTOR/Notch1 axis, thereby supporting intestinal barrier integrity. These findings highlight the capacity of the intestine to self-regulate in response to injury when sufficient metabolic resources or biosynthetic precursors are available.

## Data Availability

The raw data supporting the conclusions of this article will be made available by the authors, without undue reservation.
