# Supplementary material for: Glutamine alleviates radiation-induced intestinal injury in rats via the mTOR/Notch1 axis
Source: Front Oncol. 2026 Feb 4;16:1735401. doi: 10.3389/fonc.2026.1735401 (PMC12913147; doi:10.3389/fonc.2026.1735401)
Supplement: Supplementary file 1 [file Table1.docx]

| Antibody | Company | Country |
| --- | --- | --- |
| GAPDH Monoclonal antibody | Proteintech Group | China |
| mTOR Monoclonal antibody | Proteintech Group | China |
| p-mTOR Monoclonal antibody | Proteintech Group | China |
| S6K1 Monoclonal antibody | Proteintech Group | China |
| p-S6K1 Monoclonal antibody | Proteintech Group | China |
| 4ebp1 Monoclonal antibody | Proteintech Group | China |
| p-4ebp1 Monoclonal antibody | Proteintech Group | China |
| Notch1 Monoclonal antibody | Proteintech Group | China |
| MUC2 Monoclonal antibody | Santa | America |
| Lgr5 Monoclonal antibody | Beyotime Biotech Inc | China |
| Klf4 Monoclonal antibody | Proteintech Group | China |
| HRP-conjugated Goat Anti-Rabbit IgG | Proteintech Group | China |

Table S1 Antibodies used in this study
